# Human genetic selection on the MTHFR 677C>T polymorphism

**DOI:** 10.1186/1471-2350-9-104

**Published:** 2008-11-28

**Authors:** Álvaro Mayor-Olea, Gonzalo Callejón, Arturo R Palomares, Ana J Jiménez, María Jesús Gaitán, Alfonso Rodríguez, Maximiliano Ruiz, Armando Reyes-Engel

**Affiliations:** 1 Department of Biochemistry and Molecular Biology, University of Malaga, Malaga 29071, Spain; 2 Department of Biochemistry, Hospital Costa del Sol, Marbella (Málaga) 29603, Spain; 3 Department of Pathologic Anatomy, Hospital Materno-Infantil Carlos Haya, Malaga 29009, Spain; 4 Hospital Clínico Universitario Virgen de la Victoria Málaga 29010, Spain

## Abstract

**Background:**

The prevalence of genotypes of the 677C>T polymorphism for the MTHFR gene varies among humans. In previous studies, we found changes in the genotypic frequencies of this polymorphism in populations of different ages, suggesting that this could be caused by an increase in the intake of folate and multivitamins by women during the periconceptional period. The aim was to analyze changes in the allelic frequencies of this polymorphism in a Spanish population, including samples from spontaneous abortions (SA).

**Methods:**

A total of 1305 subjects born in the 20th century were genotyped for the 677C>T polymorphism using allele specific real-time PCR with Taqman^® ^probes. A section of our population (n = 276) born in 1980–1989 was compared with fetal samples (n = 344) from SA of unknown etiology from the same period.

**Results:**

An increase in the frequency of the T allele (0.38 vs 0.47; p < 0.001) and of the TT genotype (0.14 vs 0.24; p < 0.001) in subjects born in the last quarter of the century was observed. In the 1980–1989 period, the results show that the frequency of the wild type genotype (CC) is about tenfold lower in the SA samples than in the controls (0.03 vs 0.33; p < 0.001) and that the frequency of the TT genotype increases in the controls (0.19 to 0.27) and in the SA samples (0.20 to 0.33 (p < 0.01)); r = 0.98.

**Conclusion:**

Selection in favor of the T allele has been detected. This selection could be due to the increased fetal viability in early stages of embryonic development, as is deduced by the increase of mutants in both living and SA populations.

## Background

The methylenetetrahydrofolate reductase enzyme (MTHFR) catalyzes a reaction that produces 5-methyltetrahydrofolate (5-methylTHF), the methyl donor for homocysteine in the synthesis of methionine. The 677C>T mutation of the *MTHFR *gene has been associated with a thermolabile enzyme with decreased activity that may cause an increase in plasma homocysteine concentrations [1] when folate status is poor. This polymorphism is one of the most widely studied clinically relevant polymorphisms in humans, as it is related to cardiovascular disease [2] and neural tube defects (NTD; 601634) [3].

A large number of studies have provided a broad overview of the prevalence of the 677C>T polymorphism in different human populations, showing that the distribution of frequencies is diverse [4]. These differences have been also observed between groups of different ages in the same Spanish population (older and younger than 24 years) [5] and in a Swiss population (older and younger than 60 years) [6], as well as in a Japanese population [7].

In some populations, such the Toscanians in Italy [8] and Mexicans [9], the homozygous mutated genotype (TT) has reached frequencies greater than 30%. On the other hand, in Africans the frequency of the TT genotype is very low (less than 1%) [10,11], but, in African-Americans, it has already reached 2% [12]. Studies based on the distribution of genotypic and allelic frequencies of the 677C>T polymorphism and the 1298A>C polymorphism in the *MTHFR *gene in Israeli, Japanese and Ghanaian Africans populations [13] concluded that the 677T mutation in the *MTHFR *gene emerged as a founder haplotype with some selective advantage. Recently, preliminary evidence of genetic selection of this polymorphism related to folate intake has been reported [14].

The aim of the present study is to analyze the changes in frequencies of the 677C>T polymorphism during the 20^th ^century and particularly the evolution of the frequencies during the decade of 1980–1989, by comparing the genotype frequencies between living subjects born in this period versus samples of spontaneous abortions (SA) that occurred during in the same time period.

## Methods

### Subjects

This study was approved by the Ethics Committee at the University Hospital "Virgen de la Victoria" (Málaga). One of the study groups consisted of 344 fetal tissue samples from SA, obtained from the Department of Pathology of the University Hospital Carlos Haya (Málaga). These samples were selected after checking the clinical history and by the inclusion criteria of containing histologically confirmed fetal tissue collected in the 1980s from SA at less than 3 months (11 ± 1.70 week) and of unknown etiology. These fetal samples were compared with a control population of 276 subjects born in the 1980s with an average age of 22 ± 4.58.

Another population of subjects born in the south of Spain in the 20th century were genotyped (1305 subjects, 697 women and 608 men) and divided into four groups according to birth date: 1900 to 1925 (n = 206); 1926 to 1950 (n = 320), 1951 to 1975 (n = 408), 1976 to 2000 (n = 371). Individuals were selected randomly from different areas of the province of Malaga, in southern Spain, and from different social statuses to avoid a selection bias. All the selected individuals were Caucasian and residents of the study area. The parents of all subjects included in the study were also Caucasian and born in Spain. The possibility of a founder effect or genetic drift was investigated and rejected. All the selected individuals were also genotyped for an insertion/deletion polymorphism in the angiotensin converting enzyme (ACE) gene and/or the 2756A>G polymorphism in the methionine synthase gene (MTR), in order to determine whether or not our adult and young populations were genetically homogeneous. No significant differences were observed in allelic or genotypic frequencies for these genes between the different groups.

The population studied was randomly selected according to age. Subjects 0–12 years old were selected from dried blood spots from neonatal screening papers; subjects 10–24 years old were recruited from students in primary and secondary schools and in university; subjects 25–50 years old and >51 years old were recruited using their Andalusia Health Service identity cards. After approval by the University Hospital Ethical Committee, all the subjects were contacted, and, from those whose written consent was obtained, 10 ml of blood was taken. The investigation in this study conforms to the principles outlined in the Declaration of Helsinki.

### Genetic analysis

The fetal samples were extracted from the archived formalin-fixed, paraffin-embedded tissue sections. Genomic DNA was extracted from fetal tissue using the method described by Coombs et al. (1999) [15]. Genomic DNA of the second and third groups was extracted from peripheral leukocytes using the AquaPure Genomic DNA Blood Kit (Bio-Rad).

Genotyping was performed using Real Time PCR with allele specific Taqman^® ^probes and primers described by Ulvik et al. (2001) [16] and the following optimized protocol for 45 cycles: 10 s – 94°C, 40 s – 54°C, 15 s – 72°C. The PCR mix (25 μl total volume) consisted of 5 μl of genomic DNA, 0.5 μl of sense primer, 0.62 μl of anti-sense primer, 0.85 μl Taqman^® ^probe FAM, 0.43 μl Taqman^® ^probe TET, 20 μl PCR-buffer iQ-SupermixTM (Bio-Rad) (containing 100 mM KCl, 40 mM Tris-HCl, (pH 8.4) 1.6 mM dNTP (dATP, dCTP, dGTP and dTTP), iTaq^® ^polymerase (50 units/mL) and 6 mM MgCl2) and 17.75 μl H2O.

### Statistical and mathematical analysis

All samples were genotyped, and the allelic and genotypic frequencies were compared. Differences were analyzed statistically using the chi-square test or Fisher's exact test. Correlations are expressed using Pearson's coefficient (r).

Compliance of genotype distributions with Hardy-Weinberg (HW) equilibrium was evaluated by chi-square analysis. For all tests, a p-value < 0.05 was considered to be statistically significant. Values are expressed as the mean ± SD.

The genetic selection model was calculated for the evolution of the 677C>T genotypes. The genetic selection could be classified as codominant or incompletely dominant and directional with the heterozygous genotype having an intermediate fitness. For this kind of selection, the most appropriate mathematical model is dq = [spq(2hp+q-h)]/[p^2 ^+ 2pq(1-hs) + q^2 ^× (1-s)], where dq is the change of frequency of the allele with lower fitness, s is the fraction of that genotype lost to selection, h is the degree of dominance (between 0, for no dominance and 1, for complete dominance), and p is the frequency of the allele with higher fitness.

## Results

We analyzed the genotype frequencies of the 677C>T polymorphism in a population born during the 20th century. A total of 1305 subjects were divided into four groups of 25 years according to birth date. The genotype frequencies were compared between the four quarters of the century and showed very significant changes (p < 0.001) in the group born in the last quarter of the 20th century (1976–2000), when compared to any of the other groups. The changes show a decrease of the CC genotype and an increase of the TT genotype in the last 25 years of the 20th century. (Table 1)

**Table 1:** Distribution of C677T genotype frequencies in the 20th century.

	**Frequencies by date of birth**							
	
**C677T-*MTHFR*** **Genotype-Allele**	***1900–1925*** ***(n = 206)***		***1926–1950*** ***(n = 320)***		***1951–1975*** ***(n = 408)***		***1976–2000*** ***(n = 371)***	
	***%***	***n***	***%***	***n***	***%***	***n***	***%***	***n***
**CC**	0.393	81	0.394	126	0.37	151	0.299*	111
**CT**	0.495	102	0.488	156	0.49	200	0.461	171
**TT**	0.112	23	0.119	38	0.14	57	0.24**	89
**C allele**	0.641	264	0.638	408	0.615	502	0.53**	393
**T allele**	0.359	148	0.363	232	0.385	314	0.47**	349

Note: *p < 0.05; **p < 0.01 (significance values in the last quarter of the century are relative to any other quarter of the century)

**Figure 1:**
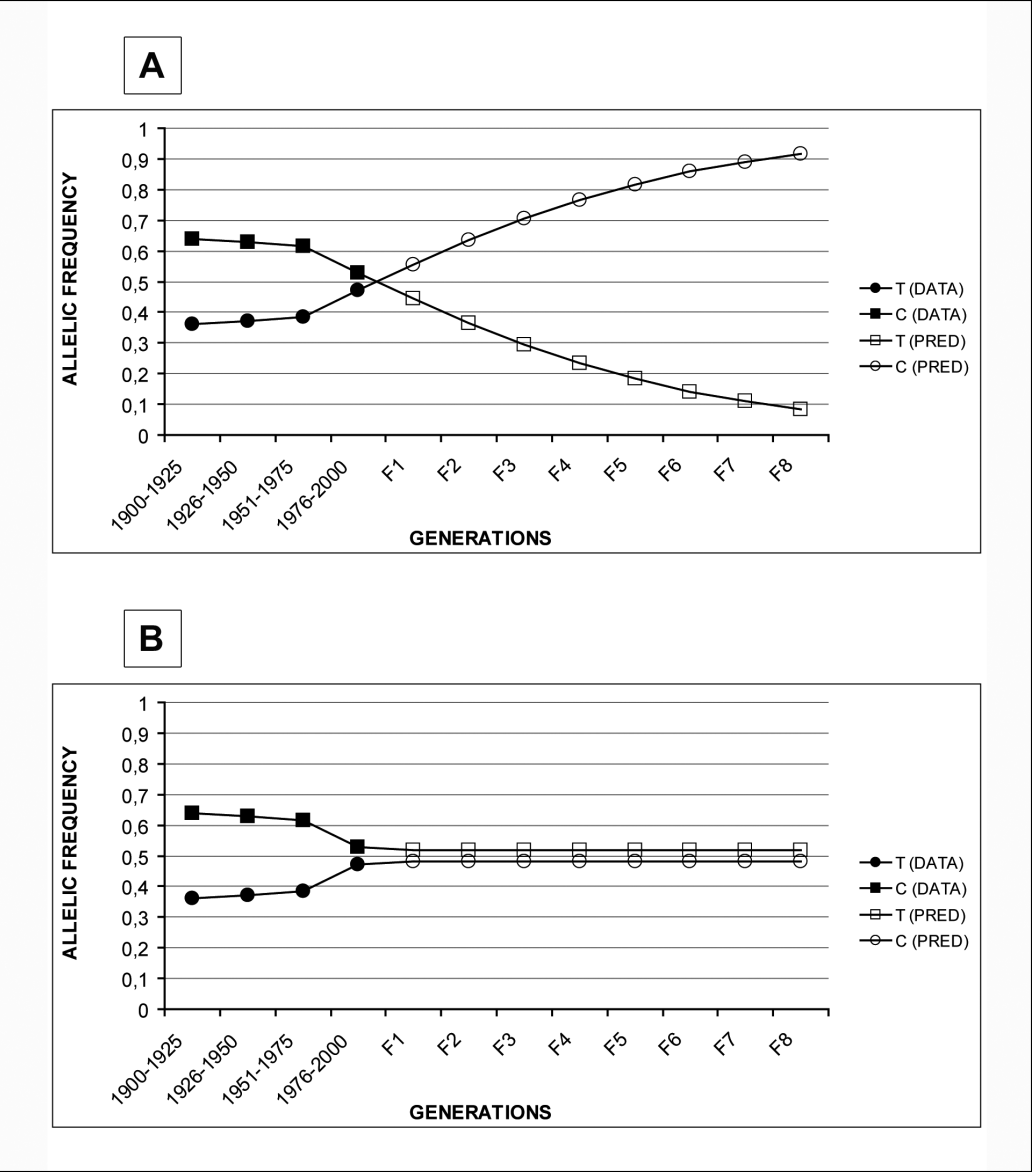
**Hypothetical evolution of the allelic frequencies of mthfr 677c>t polymorphism**. Panel A represents the observed evolution of the allelic frequencies in the 20th century (DATA) and the hypothetical evolution predicted by the mathematical selection model (calculated fitness: s = 0.5) (PRED). Panel B represents the evolution of the allelic frequencies in the 20th century (DATA) and the hypothetical evolution based on an allelic balance dependent on folate abundance conditions.

Considering that each 25 year period corresponds to a generation, allelic frequencies did not change during the first 75 years of the century (HW equilibrium). However, we found that allelic and genotypic frequencies for the 677C>T polymorphism in the last quarter of the century are significantly different compared to the previous generation (1951–1975). The genotype frequencies in the last quarter of the century are not the expected by a HW calculation using the allelic frequencies of the previous generation. This could be described as a consequence of genetic selection found in this population, in the absence of other causes. Applying the mathematical model described above to our population, the calculated fitness (s) is 0.5, and it can be predicted that both alleles will be approximately at a frequency of 50% in the next generation and allele T will be at 90% after seven generations (Figure 1A). Another possibility is that a scenario could be predicted in which both alleles will have frequencies of about 50% in the next generation and that they will maintain this stability while conditions remain unchanged. (Figure 1B)

The comparison of the genotype frequencies between a group of fetal samples from SA that occurred during the 1980–1989 decade and living subjects born in the same decade showed significant differences in genotype frequencies (p < 0.001). CC genotypes were almost absent in abortion samples compared to living subjects (0.03 vs 0.33), while CT and TT genotypes were overrepresented in the same group. When 3-year periods are studied in the decade, we detected a significant increase of the mutated subjects during the decade (CT p < 0.05; TT p < 0.01). Allele frequencies showed the same pattern (p < 0.05). Controls showed the same tendency but without statistical significance. (Table 2)

**Table 2:** Allelic and genotypic frequency comparison between fetal and control groups.

**C677T-*MTHFR*** **Genotype-Allele**	**Frequencies by date of birth**					
	**82–84**		**85–87**		**88–90**	
***Fetal Group***	*(n = 110)*		*(n = 129)*		*(n = 105)*	
	
	%	n	%	n	%	n
	
**CC**	0.073**	8	0.008**	1	0.019**	2
**CT**	0.727**	80	0.705**	91	0.6*#	63
**TT**	0.2	22	0.287	37	0.381##	40
**C allele**	0.436**	96	0.36**	93	0.319**#	67
**T allele**	0.564**	124	0.64**	165	0.681**#	143
						
***Control Group***	*(n = 88)*		*(n = 93)*		*(n = 95)*	
	
	%	n	%	n	%	n
	
**CC**	0.33	29	0.301	28	0.305	29
**CT**	0.477	42	0.452	42	0.421	40
**TT**	0.193	17	0.247	23	0.274	26
**C allele**	0.568	100	0.527	98	0.516	98
**T allele**	0.432	76	0.473	88	0.484	92

Note: *p < 0.05; **p < 0.01 (significant values relative to controls). #p < 0.05; ##p < 0.01 (significant values relative to the 1982–1984 period)

The evolution of genotype frequencies during the 1980–1989 decade of the TT genotypes correlates well in both living populations as well as fetal samples r = 0.98 (p = 0.11).

## Discussion

Different reports show that the prevalence of the 677C>T polymorphism of the *MTHFR *gene differs dramatically among human populations. Evidence of this dynamism can be observed in many reports: frequency variations between populations that are geographically very close, even in the same country [8]; changes found in the same race or ethnic group such as Africans [10,11] and African-Americans [12]; the high prevalence of the 677C>T poymorphism in populations with special nutritional features such as Mexicans [9] and Japanese [13]; and changes in frequencies between generations of the same population, as has been observed in Spain [5], Switzerland [6] and Japan [7].

There are numerous interpretations of this great diversity, and most tend to be related to adaptation to external conditions such as climate or nutritional status. Dependence of folate degradation on skin pigmentation [17], nutritional habits or human intervention periconceptional periods could explain this genetic variation. Definitely, external factors in combination with different levels of MTHFR enzyme activity, conditioned by polymorphisms, could influence the fetal viability of certain genotypes.

In 1998, we suggested the possibility of genetic selection in Spain in favor of the mutants of the 677C>T polymorphism in the *MTHFR *gene based on the fact that treatment with vitamins and folates during pregnancy increased the viability of fetuses with the TT homozygous genotype. This hypothesis was based on the increase in the number of mutated individuals found in our population since the mid-1970s [5] and the coincident increased intake of vitamins and folate by pregnant women in Spain [18,19]. In 2002, a new study found changes in genotype frequencies for the 677C>T and 1298A>C polymorphisms in different age groups. Total homocysteine (tHcy) levels in plasma were also analyzed according to the different genotype interactions [20]. That study hypothesized about fetal viability and about a genetic selection model on the basis of non-linkage disequilibrium between both polymorphisms. Recently, a study with fetal and control populations showed the strong influence of these polymorphisms, though mainly of the 677C>T polymorphism, on spontaneous early abortion [21]. In the present study, significant changes in allelic and genotypic frequencies are detected, as is Hardy-Weinberg disequilibrium, at the 677C>T polymorphism. We hypothesize that there is a dynamic process of genetic selection that favors the T allele. This process of selection started during the last quarter of the 20th century, during which the frequency for mutant homozygous (TT) rose significantly from 14% to 24%. We propose that this increase in mutants is due to the inclusion of an external factor that enhances mutant fetal viability.

If we apply the mathematical model for dynamic selection developed for diploid organisms with sexual reproduction, the T allele could reach to 90% in seven generations in our population (Figure 1A). However, this model assumes selection in a constant environment that applies to all individuals in the population studied. In our case, we suggest that the external factor is related to an increase in folate and vitamin intake in women in periconceptional period and does not affect to all individuals [18,19]. We assume that prediction of a classic selection model in this case is only theoretical.

On the basis of a competition between alleles in which an environmental factor favors one allele versus the other, the final result would be that predicted by the previous mathematical model. However in this case, the environment is not selecting against the wild type allele but rather allowing the survival of more mutated alleles. Therefore, the expected result would be not a systematic increase of the mutated allele but the creation of an allelic balance dependent on vitamin and folate abundance conditions. In this case, the mutation would have a lower influence on fetal viability (Figure 1B).

The results showed an increase in mutated genotypes (CT and TT) and a strong protection against abortion by the wild type genotype (CC), which is practically non-existent in the SA group. The frequency of the CC genotype shows no change over the decade studied (1980–1989), which indicates that folate does not exert a visible effect on this genotype. However, the frequency of the mutated allele increases during this decade, especially in fetuses from abortions, and this increase correlated with the increase of the T allele in the control population. This finding suggests that the effect of folate is crucial to viability during the early stages of embryonic development, but, even with folate, not all embryos will survive until birth.

In this population, the mutant allele with lower enzymatic activity has higher fitness than the wild type. In the folate cycle, it can be observed that 5,10-methyleneTHF availability may be important. 5,10-methyleneTHF is the substrate for several reactions in the cycle, but two of them (5-methylTHF and thymidilate synthesis) might be essential for embryo development in folate deficiency conditions.

In both cases, complete or limited MTHFR activity will produce higher or lower 5,10-methyleneTHF availability, which might be an essential factor for embryo development, such that a greater folate levels can compensate the lower enzymatic activity of the mutant.

The implications of this polymorphism in nucleotide synthesis have not yet been determined, but certain data, such as high levels of uric acid found in mutated subjects [22,23], suggest that there are different turnover rates associated with different polymorphisms.

## Conclusion

We suggest that there is genetic selection in our population for the T allele of the *MTHFR *– 677C>T polymorphism, whose origin could be an increase in fetal viability during the early stages of embryonic development because of an increase in folate and vitamin intake by women in the periconceptional period that began to be established in Spain in the last quarter of the 20th century [18,19]. Higher frequencies for the T allele and TT genotype in our population are observed in the living and SA populations.

## Competing interests

The authors declare that they have no competing interests.

## Authors' contributions

AMO performed the statistical analysis, helped to draft the manuscript and revised it for publication. GC is the corresponding author, participated in the acquisition of samples and carried out the genotyping. ARP carried out the bibliographic search and helped to draft the manuscript. AJJ participated in the selection and the processing of samples. MJG coordinated the laboratory work and selected the genotyping method. AR selected the control subjects and designed the consent form. MR helped in the interpretation of data and tables performance. ARE conceived the study and is the guarantor of this work and the general coordinator. All authors read and approved the final manuscript.

## Pre-publication history

The pre-publication history for this paper can be accessed here:



## References

[B1] Frosst P, Blom HJ, Milos R, Goyette P, Sheppard CA, Matthews RG, Boers GJ, den Heijer M, Kluijtmans LA, Heuvel LP van den (1995). A candidate genetic risk factor for vascular disease: a common mutation in methylenetetrahydrofolate reductase. Nat Genet.

[B2] Klerk M, Verhoef P, Clarke R, Blom HJ, Kok FJ, Schouten EG (2002). MTHFR Studies Collaboration Group. MTHFR 677C-T polymorphism and risk of coronary heart disease: a meta-analysis. JAMA.

[B3] Johanning GL, Wenstrom KD, Tamura T (2002). Changes in frequencies of heterozygous thermolabile 5,10-methylenetetrahydrofolate reductase gene in fetuses with neural tube defects. J Med Genet.

[B4] Wilcken B, Bamforth F, Li Z, Zhu H, Ritvanen A, Redlund M, Stoll C, Alembik Y, Dott B, Czeizel AE, Gelman-Kohan Z, Scarano G (2003). Geographical and ethnic variation of the 677C-T allele of 5,10 methylenetetrahydrofolate reductase (MTHFR): findings from over 7000 newborns from 16 areas worldwide. J Med Genet.

[B5] Muñoz-Moran E, Dieguez-Lucena JL, Fernandez-Arcas N, Peran-Mesa S, Reyes-Engel A (1998). Genetic selection and folate intake during pregnancy. Lancet.

[B6] Todesco L, Angst C, Litynski P, Loehrer F, Fowler B, Haefeli WE (1999). Methylenetetrahydrofolate reductase polymorphism, plasma homocysteine and age. Eur J Clin Invest.

[B7] Matsushita S, Muramatsu T, Arai H, Matsui T, Higuchi S (1997). The frequency of the methylenetetrahydrofolate reductase-gene mutation varies with age in the normal population. Am J Hum Genet.

[B8] Abbate R, Sardi L, Pepe G, Marcucci R, Brunelli T, Prisco D, Fatini C, Capanni M, Simonetti L, Gensini GE (1998). The high prevalence of thermolabile 5–10 methylenetetrahydrofolate reductase (MTHFR) in ltalians is not associated to an increased risk for coronary artery disease (CAD). Thromb Haemast.

[B9] Mutchinick OM, López MA, Luna L, Waxman J, Babinsky VE (1999). High prevalence of the thermolabile methylenetetrahydrofolate reductase variant in Mexico: a country with a very high prevalence of neural tube defects. Mol Genet Metab.

[B10] Rajkovic A, Mahomed K, Rozen R, Malinow MR, King IB, Williams MA (2000). Methylenetetrahydrofolate reductase 677 C--> T polymorphism, plasma folate, vitamin B(12) concentrations, and risk of preeclampsia among black African women from Zimbabwe. Mol Genet Metab.

[B11] Guéant-Rodriguez RM, Guéant JL, Debard R, Thirion S, Hong LX, Bronowicki JP, Namour F, Chabi NW, Sanni A, Anello G, Bosco P, Romano C, Amouzou E, Arrieta HR, Sánchez BE, Romano A, Herbeth B, Guilland JC, Mutchinick OM (2006). Prevalence of methylenetetrahydrofolate reductase 677T and 1298C alleles and folate status: a comparative study in Mexican, West African, and European populations. Am J Clin Nutr.

[B12] McAndrew PE, Brandt JT, Pearl DK, Prior TW (1996). The incidence of the gene for thermolabile methylene tetrahydrofolate reductase in African Americans. Thromb Res.

[B13] Rosenberg N, Murata M, Ikeda Y, Opare-Sem O, Zivelin A, Geffen E, Seligsohn U (2002). The frequent 5,10-methylenetetrahydrofolate reductase C677T polymorphism is associated with a common haplotype in whites, Japanese, and Africans. Am J Hum Genet.

[B14] Lucock M, Yates Z, Ng X, Veysey M, Blades B, Travers C, Lewis P, Sturm J, Roach P (2008). Preliminary Evidence for Genetic Selection of *677T*-MTHFR by Natural Annual Cycle of Folate Abundance. J Nutrigenet Nutrigenomics.

[B15] Coombs NJ, Gough AC, Primrose JN (1999). Optimisation of DNA and RNA extraction from archival formalin-fixed tissue. Nucleic Acids Res.

[B16] Ulvik A, Ueland PM (2001). Single nucleotide polymorphism (SNP) genotyping in unprocessed whole blood and serum by real-time PCR: application to SNPs affecting homocysteine and folate metabolism. Clin Chem.

[B17] Cordain L, Hickey MS (2006). Ultraviolet radiation represents an evolutionary selective pressure for the south-to-north gradient of the MTHFR 677TT genotype. Am J Clin Nutr.

[B18] Salvador J, Martinez-Frias ML, Rodriguez-Pinilla E (1989). Consumo de medicamentos por la mujer embarazada en España: perfil de una muestra de la población en los años 1976–1986.

[B19] Rodriguez-Pinilla E, Bermejo-Sánchez E, Martinez-Frias ML (1993). Medicamenrtos de uso más frecuente durante la gestación.

[B20] Reyes-Engel A, Muñoz E, Gaitan MJ, Fabre E, Gallo M, Dieguez JL, Ruiz M, Morell M (2002). Implications on human fertility of the 677C→T and 1298A→C polymorphisms of the MTHFR gene: consequences of a possible genetic selection. Mol Hum Reprod.

[B21] Callejón G, Mayor-Olea A, Jiménez AJ, Gaitán MJ, Palomares AR, Martínez F, Ruiz M, Reyes-Engel A (2007). Genotypes of the C677T and A1298C polymorphisms of the MTHFR gene as a cause of human spontaneous embryo loss. Hum Reprod.

[B22] Motti C, Gnasso A, Bernardini S, Massoud R, Pastore A, Rampa P, Federici G, Cortese C (1998). Common mutation in methylenetetrahydrofolate reductase. Correlation with homocysteine and other risk factors for vascular disease. Atherosclerosis.

[B23] Zuo M, Nishio H, Lee MJ, Maejima K, Mimura S, Sumino K (2000). The C677T mutation in the methylene tetrahydrofolate reductase gene increases serum uric acid in elderly men. J Hum Genet.

